# Urate is closely linked to white matter integrity in multiple system atrophy

**DOI:** 10.1002/acn3.51073

**Published:** 2020-06-26

**Authors:** Han Soo Yoo, Seok Jong Chung, Yang Hyun Lee, Byoung Seok Ye, Young H. Sohn, Hunki Kwon, Phil Hyu Lee

**Affiliations:** ^1^ Department of Neurology Yonsei University College of Medicine Seoul South Korea; ^2^ Department of Neurology Yale University School of Medicine New Haven Connecticut USA; ^3^ Severance Biomedical Science Institute Yonsei University College of Medicine Seoul South Korea

## Abstract

**Objective:**

We aimed to investigate the association of the serum urate level with cortical thickness and white matter integrity in multiple system atrophy (MSA).

**Methods:**

We recruited 75 MSA patients and 42 controls who underwent brain MRI and measured serum urate level at baseline. Using cortical thickness and tract‐based spatial statistics analyses, we investigated the correlation between serum urate levels and cortical thickness or diffusion tensor imaging (DTI) measures in controls and MSA patients. Interaction effects were analyzed to find different patterns of correlation according to sex and clinical subtype. We evaluated the relationship between serum urate levels, DTI measures, and total UMSARS score, using path analysis.

**Results:**

Serum urate levels showed a positive correlation with FA values in the corpus callosum and a negative correlation with MD values in widespread regions including cerebellar, brainstem, and cerebral white matter in patients with MSA. Both sexes showed a negative correlation between serum urate levels and MD values without significant interaction effect. In subgroup analysis according to subtype, patients with cerebellar subtype showed a negative correlation. Serum urate levels did not correlated with cortical thickness. Path analysis showed that MD values in middle and inferior cerebellar peduncle mediated the association between serum urate level and total UMSAR score.

**Interpretation:**

The present study demonstrated that serum urate levels played a pivotal role in white matter disintegrity and clinical disability in MSA. It would provide an evidence of the role of urate as a potential neuroprotective factor against white matter neurodegeneration in MSA.

## Introduction

MSA is an adult‐onset and sporadic neurodegenerative disease that manifests as progressive autonomic failure, parkinsonism, cerebellar syndrome, and pyramidal features in various combinations.^1^ MSA is a relentlessly progressive disease with median disease duration of 7.5 years from the onset of symptoms to death.^2^ Despite extensive research and clinical trials, disease‐modifying candidates except stem cell therapy are not available.^3^ Pathologically, MSA is regarded as a unique oligodendroglial α‐synucleinopathy, and degeneration of myelin due to oligodendroglial dysfunction is a leading event in the pathogenesis of MSA, followed by neuronal death.^4^ Therefore, white matter alterations in supratentorial and infratentorial areas were extensively detected in patients with MSA, suggesting prominent dysfunction of myelin connecting multiple brain systems throughout the cerebral regions.^5^, ^6^, ^7^

Urate, a natural antioxidant, has been reported to have an association with the risk and progression of neurodegenerative diseases.^8^ A meta‐analysis reported that low serum urate levels were associated with an increased risk of MSA.^9^, ^10^ Patients with MSA having high serum urate levels had slower disease progression than those with lower levels.^11^ Although the role of serum urate in the pathogenesis of MSA and its causality are currently unknown, these studies suggest that urate may have a close link to neurodegeneration in MSA, probably via antioxidant effect.

In the present study, we hypothesized that serum urate levels may be closely associated with the alterations in gray matter thickness or white matter integrity in MSA. In the present study, using the analyses of cortical thickness and track‐based spatial statistics (TBSS), we explored the relationship between serum urate levels and cortical thickness or quantitative white matter metrics such as fractional anisotropy (FA) and mean diffusivity (MD) in patients with MSA.

## Methods

### Study design

We consecutively recruited 75 patients with MSA and 42 normal controls from the Movement Disorders outpatient clinic in the Yonsei University Health System from August 2011 to April 2018. Patients with MSA were diagnosed with probable MSA, parkinsonian subtype (MSA‐P), or cerebellar subtype (MSA‐C), based on the diagnostic criteria for MSA.^12^ All patients had undergone the Unified MSA Rating Scale (UMSARS) part I to IV, Korean version of the Mini‐Mental State Examination (K‐MMSE), brain magnetic resonance imaging (MRI), and either *N*‐(3‐[^18^F]fluoropropyl)‐2*β*‐carbomethoxy‐3*β*‐(4‐iodophenyl) nortropane (FP‐CIT) positron emission tomography (PET) or ^18^F‐fluorodeoxyglucose (FDG) PET scan within 6 months. UMSARS was performed to assess the disease severity at the time of MRI acquisition. Total UMSARS score was defined as the sum of UMSARS‐I (historical), II (motor examination), and IV (global disability scale) subscores. All enrolled patients had a minimum K‐MMSE score of 24. The diagnosis of MSA was supported by structural and/or functional imaging evaluations in all patients,^12^ including atrophy on MRI of the putamen, middle cerebellar peduncle, pons, or cerebellum; hypometabolism on FDG PET scan in the putamen, brainstem, or cerebellum; and presynaptic nigrostriatal dopaminergic denervation on FP‐CIT PET scan. None of the patients had mutations in the *SCA* 1, 2, 3, 6, 7, 8, and 17 genes. We excluded the following patients with: (1) focal brain lesions, severe white matter hyperintensities, multiple lacunae in the basal ganglia, or hydrocephalus on brain MRI; (2) total K‐MMSE score less than 24; (3) other major neurologic or psychiatric illnesses; (4) diseases affecting purine turnover including chronic renal disease, myeloproliferative disease, or chronic hemolytic anemia; (5) a history of heavy alcohol consumption or vegetarianism; and (6) antigout or diuretics medication. All normal controls enrolled in this study had no active neurological disorders and normal cognitive function according to the K‐MMSE (scores ≥ 24). Additionally, body mass index (kg/m^2^) and history of hypertension, diabetes mellitus, hyperlipidemia, cardiac disease, ischemic stroke, and smoking were obtained in patients and controls.

The present study was approved by the Institutional Review Board of Yonsei University College of Medicine. Written informed consent was obtained from all patients and healthy controls who participated in this study.

### Measurement of serum urate level

Urate level was measured in serum samples collected on the morning of MRI acquisition day after fasting overnight because the serum urate level could be affected by diurnal variation and food ingestion.^13^ The level of serum urate was determined by an enzymatic colorimetric method using an automatic analyzer (Hitachi 7600; Hitachi, Tokyo, Japan).

### Neuroimaging acquisition

We acquired high‐resolution T1‐weighted MRI data and diffusion tensor imaging (DTI) from all participants using a Philips 3T scanner (Philips Intera, Philips Medical System). A detailed description is provided in Method S1.

### Processing of DTI data

The Functional MRI of Brain (FMRIB) Software Library (FSL) (http://www.fmrib.ox.ac.uk/fsl) was used to preprocess the DTI data.Detailed description is provided in Method S2.

### Processing of high‐resolution T1‐weighted MR data

We used the same methodology that we employed in a previous study,^14^ and a detailed description is provided in Method S3.

### Analyses of cortical thickness and TBSS

While comparing the localized differences in the DTI measures (FA and MD) and cortical thickness between normal controls and MSA, we performed voxel‐wise group comparison of individual skeleton images using a nonparametric permutation test, after controlling for age and sex in the comparison between the MSA patients and the normal controls and for age, disease duration, and total UMSARS score in the comparison between male and female MSA patients.

To investigate the correlation between serum urate levels and DTI measures or cortical thickness, we performed a voxel‐wise correlation analysis using a nonparametric permutation test. In all MSA patients and patients with MSA‐C and MSA‐P, we used age, sex, disease duration, and total UMSARS score as covariates. In male and female MSA patients, we used age, disease duration, and total UMSARS score as covariates. We also examined the interaction between the serum urate and sex and between the serum urate and clinical subtype to investigate the differential effect of the serum urate on the DTI measures according to sexes and clinical subtype.

The null distribution of all permutation tests in this study was built over 5000 permutations. We used the threshold‐free cluster enhancement with the two‐dimensional parameter settings^15^ to avoid an arbitrary threshold of the initial cluster formation. Corrections for multiple comparisons were performed using family‐wise error rate in diffusion tensor imaging analysis and random field theory in cortical thickness analysis respectively. The threshold was set at corrected *P* < 0.05.

### Statistical analyses

The baseline demographic and clinical characteristics of patients with MSA were analyzed, using Student’s t‐tests for continuous variables, while Pearson’s *χ* ^2^ tests were used to analyze categorical variables. As there were significant relationship between the serum urate, DTI measures, and total UMSARS score, we performed path analyses to evaluate whether DTI measures mediated the association between the serum urate and total UMSARS score after controlling for age, sex, and disease duration. Among the regions where there were significant correlation between the serum urate and DTI measures in all MSA patients, those showing significant association with total UMSARS score were selected as predictors for path analyses. We used a bootstrapping method with 500 resamples to derive the 95% confidence intervals and standard errors. The data were analyzed using SPSS software 23 (IBM Corporation, Armonk, NY) and AMOS software 23 (IBM Corporation, Armonk, NY). *P* values < 0.05 were considered significant.

## Results

### Demographic and clinical characteristics

The baseline demographic and clinical characteristics of the study subjects are summarized in Table 1. Patients with MSA had lower serum urate levels and K‐MMSE scores than the control subjects. Male patients with MSA had lower K‐MMSE scores than the male controls, while female patients with MSA had lower K‐MMSE scores and serum urate levels than the female controls. Male patients with MSA had significantly higher levels of urate than the female patients with MSA (5.4 ± 1.3 vs. 3.6 ± 0.9, *P* < 0.001). Other characteristics were not significantly different between the MSA patients and the control groups and between the male and the female patient groups.

**Table 1:** Demographic characteristics of patients with multiple system atrophy and healthy control.

	MSA, all	NC, all	*P*‐value^1^	Male MSA	Male control	*P*‐value^2^	Female MSA	Female control	*P*‐value^3^	*P*‐value^4^
Number of subjects	75	42		42	22		33	20		
Age at onset, y	58.5 ± 8.7	61.5 ± 6.0	0.094	57.7 ± 9.2	60.5 ± 6.7	0.122	59.5 ± 7.9	60.4 ± 5.0	0.613	0.381
Disease duration, y	2.1 ± 1.3	NA	–	1.9 ± 1.1	NA	–	2.3 ± 1.5	NA	–	0.140
Serum urate, mg/dL	4.6 ± 1.5	5.2 ± 1.3	0.044	5.4 ± 1.3	5.8 ± 1.4	0.212	3.6 ± 0.9	4.4 ± 0.6	0.001	<0.001
Body mass index, kg/m^2^	23.9 ± 3.2	24.7 ± 3.3	0.241	24.6 ± 2.8	25.2 ± 3.4	0.436	23.2 ± 3.5	24.1 ± 3.2	0.321	0.058
K‐MMSE	26.7 ± 2.1	29.0 ± 0.9	0.009	27.2 ± 2.0	29.1 ± 1.0	0.001	26.1 ± 2.2	28.9 ± 0.9	0.001	0.124
Clinical subtype, *n* (%)										0.225
MSA‐P	35 (46.7)	NA	–	17 (40.5)	NA	–	18 (54.5)	NA	–	
MSA‐C	40 (53.3)	NA	–	25 (59.5)	NA	–	15 (45.5)	NA	–	
Total UMSARS score	35.3 ± 17.0	NA	–	35.4 ± 13.5	NA	–	41.2 ± 17.8	NA	–	0.069
Part I	16.8 ± 8.1	NA	–	16.1 ± 6.6	NA	–	18.5 ± 8.5	NA	–	0.247
Part II	17.4 ± 8.7	NA	–	15.9 ± 6.1	NA	–	20.4 ± 8.5	NA	–	0.113
Part IV	1.9 ± 1.0	NA	–	1.8 ± 0.8	NA	–	2.0 ± 1.0	NA	–	0.346
Vascular risk factors, *n* (%)										
Hypertension	21 (28.0)	15 (35.7)	0.386	12 (28.6)	9 (40.9)	0.104	9 (27.3)	6 (30.0)	0.775	0.962
Diabetes mellitus	14 (18.7)	5 (11.9)	0.341	9 (21.4)	3 (13.6)	0.126	5 (15.2)	2 (10.0)	0.635	0.255
Dyslipidemia	10 (13.3)	8 (19.0)	0.411	6 (14.3)	3 (13.6)	0.685	4 (12.1)	5 (25.0)	0.070	0.440
Cardiac disease	3 (4.0)	5 (11.9)	0.104	2 (4.8)	4 (18.2)	0.199	1 (3.0)	1 (5.0)	0.278	0.207
Ischemic stroke	3 (4.0)	0 (0.0)	0.189	2 (4.8)	0 (0.0)	0.217	1 (3.0)	0 (0.0)	0.217	0.207
Current smoking	10 (13.3)	11 (26.2)	0.082	8 (19.0)	9 (40.9)	0.060	2 (6.1)	2 (10.0)	0.599	0.170

Values are expressed as mean ± standard deviation or number (percentage).

K‐MMSE, the Korean version of Mini‐Mental State Examination; MSA, multiple system atrophy; NC, normal controls; NA, not applicable; RBD, rapid eye movement sleep behavior disorder; UMSARS, Unified Multiple System Atrophy Rating System.

^1^ *P*‐value < 0.05 between MSA versus control.

^2^ *P*‐value < 0.05 between male MSA versus male control.

^3^ *P*‐value < 0.05 between female MSA versus female control.

^4^ *P*‐value < 0.05 between male MSA versus female MSA.

### Group comparison of white matter integrity and cortical thickness

When compared with the normal controls, patients with MSA had lower FA values and higher MD values in widespread brain areas including cerebellar, brainstem, and cerebral white matter (Fig. S1A). They also had lower cortical thickness in the left primary motor and premotor cortices and bilateral lateral temporal, insular, lateral and medial occipital, posterior cingulate, and retrosplenial cortices. In MSA group, neither FA and MD values nor cortical thickness were significantly different between the male and the female MSA patients (Fig. S1B).

### Correlation between serum urate levels and white matter integrity or cortical thickness in normal controls and MSA

**Figure 1:**
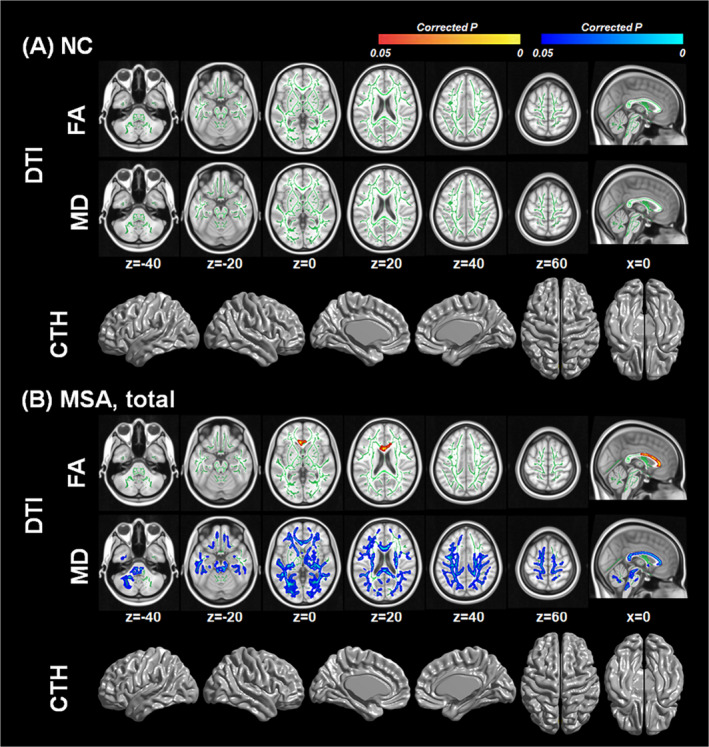
White matter integrity and cortical thickness associated with serum urate levels (A) in normal controls and (B) in patients with MSA. Results are based on general linear models using serum urate levels as a predictor and controlling for age, sex, disease duration, and total Unified MSA Rating Scale score. Corrections for multiple comparisons were performed using family‐wise error rate in diffusion tensor imaging analysis and random field theory in cortical thickness analysis respectively. Red to yellow color in corrected *P*‐map indicates regions showing significant positive correlations with serum urate levels. Blue color in corrected *P*‐map indicates regions showing significant negative correlations with serum urate levels. The threshold was set at corrected *P* < 0.05. The brain images were displayed in neurological convention.

In the control group, serum urate levels showed no correlation with FA and MD values and cortical thickness (Fig. 1A). In the MSA group, serum urate levels showed positive correlation with FA values in the corpus callosum, especially in the genu and the body portions. The urate levels in the MSA group showed negative correlation with MD values in the widespread brain areas including cerebellar, brainstem, and cerebral white matter (Fig. 1B).

### Correlation between serum urate levels and white matter integrity or cortical thickness in MSA according to sex

**Figure 2:**
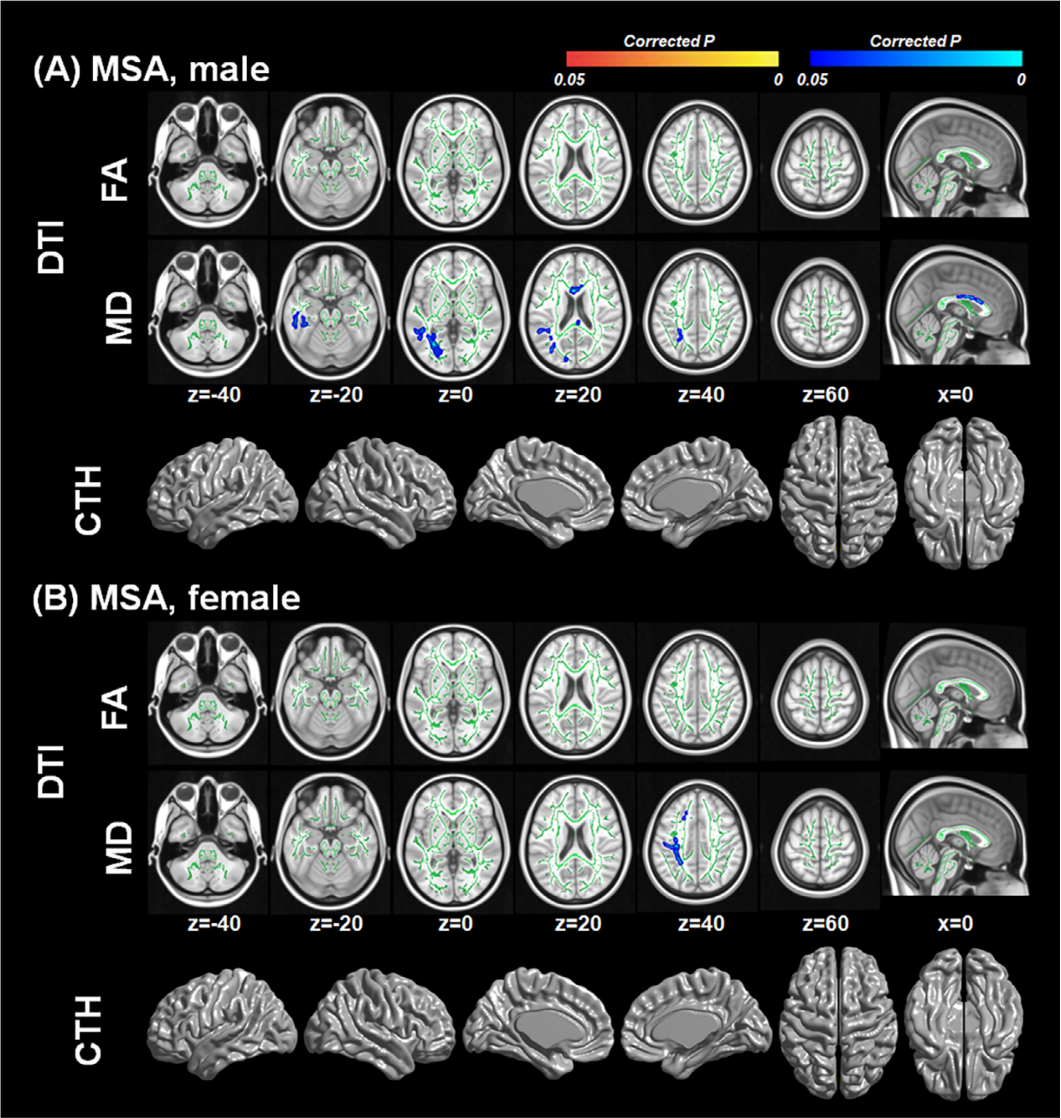
White matter integrity and cortical thickness associated with serum urate levels according to sex. General linear models were performed to investigate the correlation between serum urate levels and the diffusion tensor imaging measures or cortical thickness in (A) male and (B) female patients with MSA, after controlling for age, disease duration, and total Unified MSA Rating Scale score. Corrections for multiple comparisons were performed using family‐wise error rate in diffusion tensor imaging analysis and random field theory in cortical thickness analysis respectively. Red to yellow color in corrected *P*‐map indicates regions showing significant positive correlations with serum urate levels. Blue color in corrected *P*‐map indicates regions showing significant negative correlations with serum urate levels. The brain images were displayed in neurological convention.

Since serum urate levels were significantly different between the sexes, we performed subgroup analysis according to sexes. In male patients with MSA, serum urate levels showed negative correlation with MD values in the corpus callosum, especially the body portion, and the left temporo‐parietal white matter (Fig. 2A), but showed no correlation with FA values or cortical thickness. In female patients with MSA, serum urate levels showed negative correlation with MD values in the left fronto‐parietal white matter (Fig. 2B), but showed no significant association with FA values or cortical thickness. Interaction analysis showed that there was no urate by sex interaction effect on FA and MD values or cortical thickness.

### Correlation between serum urate levels and white matter integrity or cortical thickness in MSA according to clinical subtype

**Figure 3:**
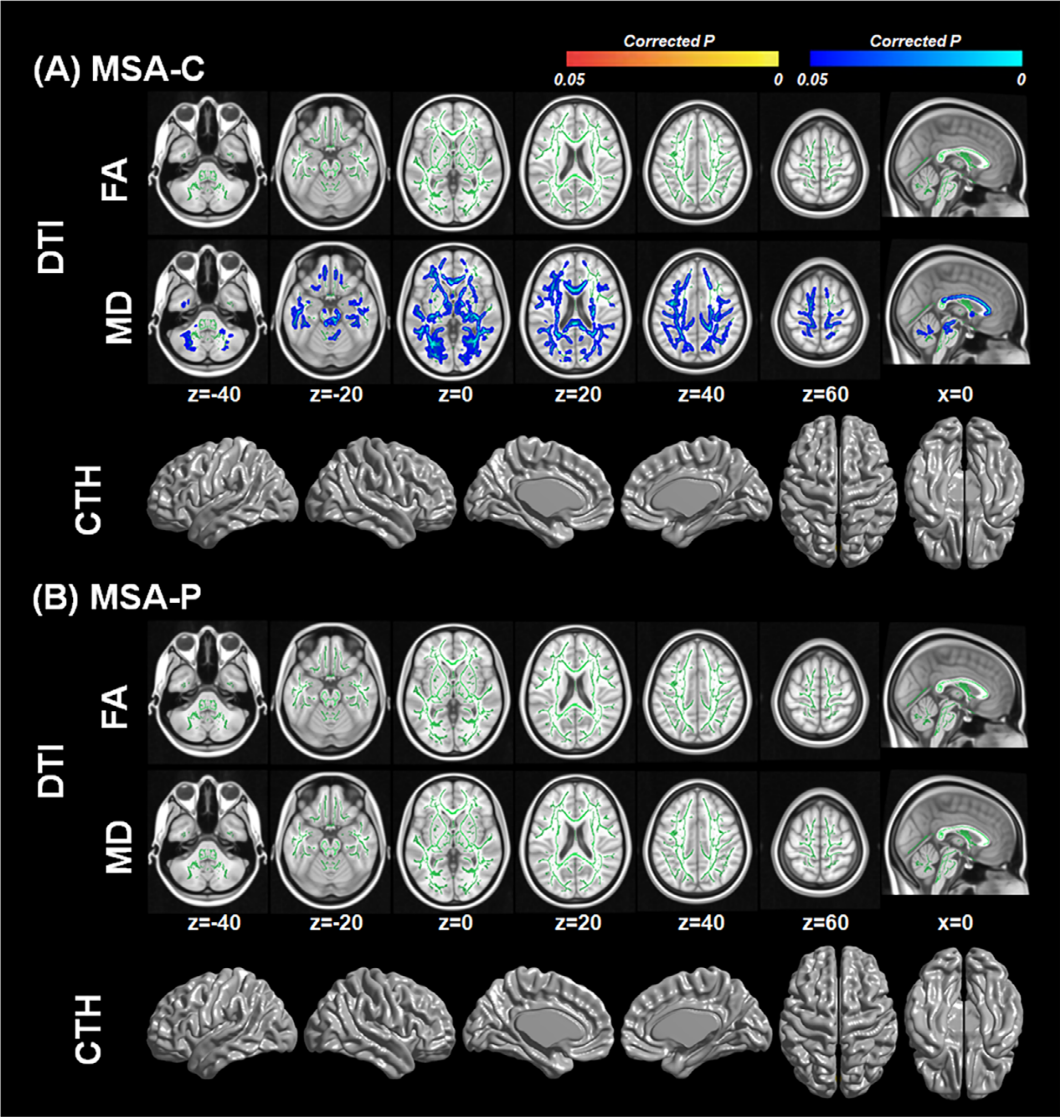
White matter integrity and cortical thickness associated with serum urate levels according to clinical subtype. General linear models were performed to investigate the correlation between serum urate levels and the diffusion tensor imaging measures or cortical thickness in MSA patients (A) with cerebellar subtype and (B) with parkinsonian subtype, after controlling for age, sex, disease duration, and total Unified MSA Rating Scale score. Corrections for multiple comparisons were performed using family‐wise error rate in diffusion tensor imaging analysis and random field theory in cortical thickness analysis respectively. Red to yellow color in corrected *P*‐map indicates white matter regions showing significant positive correlations with serum urate levels. Blue color in corrected *P*‐map indicates white matter regions showing significant negative correlations with serum urate levels. The brain images were displayed in neurological convention.

In patients with MSA‐C, serum urate levels showed negative correlation with MD values in widespread brain regions including cerebellar, brainstem, and cerebral white matter (Fig. 3A), whereas serum urate levels had no significant correlation with FA values or cortical thickness. In patients with MSA‐P, serum urate levels showed no correlation with FA and MD values or cortical thickness (Fig. 3B). Interaction analysis showed that there was no urate by MSA subtype interaction effect on FA and MD values or cortical thickness.

### Relationship among serum urate levels, DTI measures, and total UMSARS score

**Figure 4:**
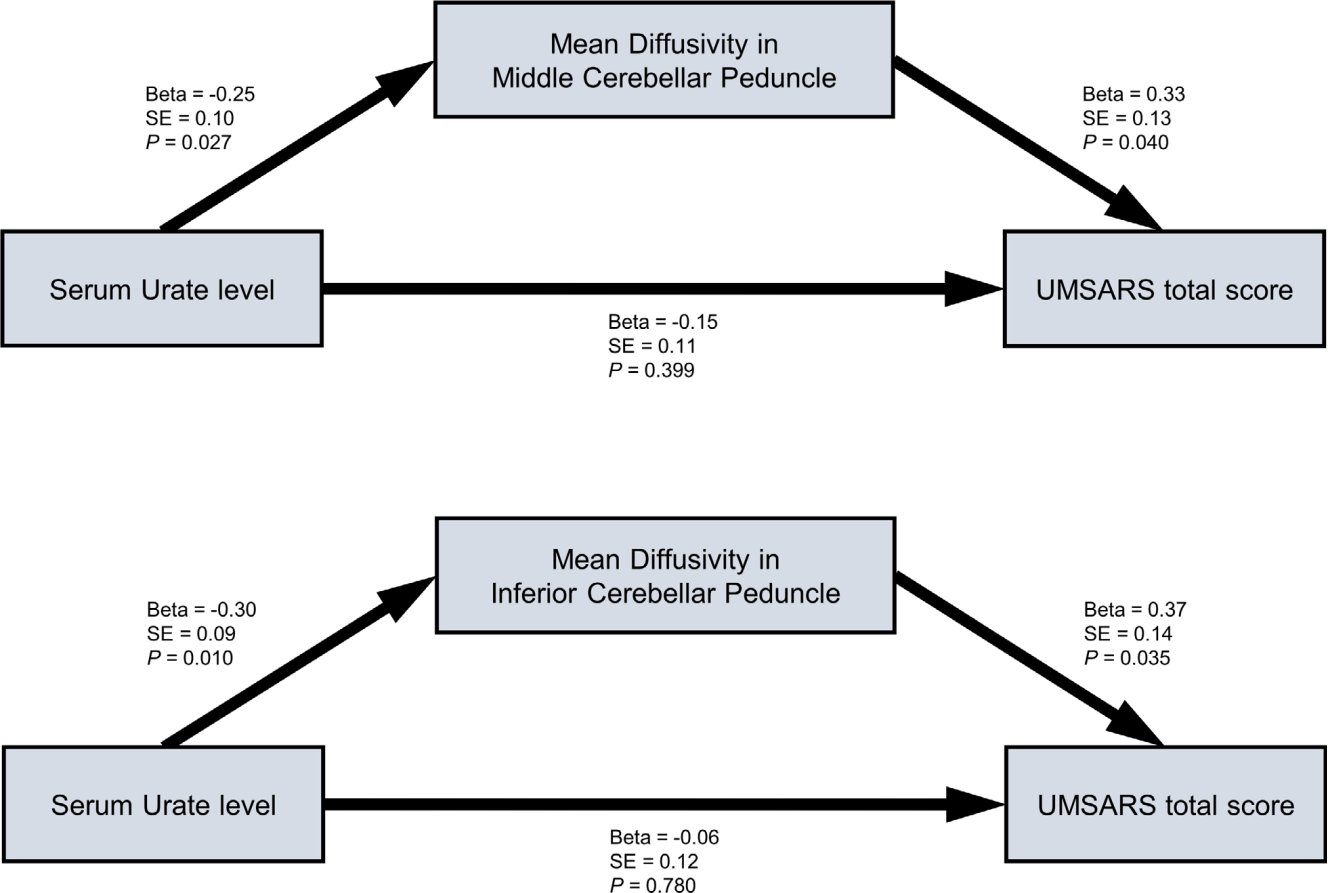
Schematic diagram of the path analyses. Serum urate level and mean diffusivity in middle cerebellar peduncle or inferior cerebellar peduncle were entered as predictors for total UMSARS score. Age, sex, and disease duration were entered as covariates.

Since there were correlations between serum urate levels, MD values in certain regions, and total UMSARS score, we performed path analyses to investigate the relationship of these variables. We set a hypothetical model, using the serum urate level as a predictor, DTI measures as a mediator, and total UMSARS score as an outcome variable. Path analyses showed that among white matter regions, mean diffusivity in middle and inferior cerebellar peduncles mediated the association between serum urate level and total UMSARS score with goodness of fit (Fig. 4; Table S1).

## Discussion

The present study assessed the association of serum urate levels with cortical thickness and white matter integrity in patients with MSA. We found that serum urate levels were not correlated with cortical thickness but with white matter integrity irrespective of sex. The association was exclusively observed in patients with MSA‐C. White matter disintegrity in middle and inferior cerebellar peduncles mediated the association between serum urate level and total UMSARS score. These findings suggest that urate is closely linked to white matter integrity and disease severity in MSA.

Accumulating evidence has suggested the role of oxidative stress in the pathogenesis of MSA. Experimental studies have demonstrated that mitochondrial dysfunction and oxidative stress are possible risk factors for triggering or exacerbating the MSA pathology.^16^, ^17^ In case–control epidemiological studies, it has been observed that occupational exposure to pesticides, insecticides, or solvents interrupting the mitochondrial electron transport and leading to oxidative stress may increase the risk of MSA.^18^, ^19^, ^20^ Low levels of reduced glutathione and high levels of iron content in the oligodendrocytes and oligodendrocyte progenitors contribute to this vulnerability to oxidative stress.^21^, ^22^ Since MSA is an oligodendroglial α‐synucleinopathy, it can be inferred that oligodendroglial cytoplasmic inclusions may increase the oxidative stress.^23^ Conversely, oxidative stress can aggravate the accumulation of α‐synuclein in oligodendrocytes.^24^ Both of these events result in impaired myelination and white matter degeneration in MSA. In the present study, we provide the evidence for the first time that higher levels of serum urate were closely associated with less severe white matter disintegrity in widespread brain areas. This result suggests a protective role of urate against white matter damage through its mediating antioxidant property.

In line with our study, previous imaging studies have consistently demonstrated white matter disintegrity in extensive supratentorial and infratentorial structures in patients with MSA.^5^, ^6^ Importantly, we found in this study that serum urate levels had a significant correlation with MD values in widespread brain regions, whereas the relationship between serum urate levels and FA values was localized in the corpus callosum. Since MD is an average of eigenvalues of three axes, it reflects the magnitude of water diffusion that is sensitive to cellular structure and edema.^25^ Meanwhile, FA quantifies the preferential direction of water diffusion along the white matter tracts that is sensitive to microstructural features including axonal density and myelination.^26^ Since urate is related to oxidative stress, which can further induce neuroinflammation,^27^ it can be inferred that urate may be more closely associated with impaired tissue barrier and increased water diffusivity around degenerated white matter tissue due to neuroinflammation than to decreased directionality of white matter tract. Among the areas with altered white matter in MSA, the corpus callosum showed significant correlation between serum urate levels and both DTI measures. It is known that the corpus callosum is a major white matter region of accumulation of glial cytoplasmic inclusions,^28^ via which spread of α‐synuclein from both hemispheres might occur.^29^ In *postmortem* tissue of MSA patients, the corpus callosum showed increased neuroinflammation with high α‐synuclein load.^30^ Thus, the corpus callosum may be a principal site of myelin dysfunction and axonal injury that is vulnerable to oxidative stress in MSA.

Several studies have reported sex differences in the neuroprotective effect of urate in various neurodegenerative disorders. In Parkinson’s disease, high urate levels have consistently been associated with lower risk among men, while the association was weaker and inconsistent among women.^31^, ^32^ Urate levels also predicted survival in men with amyotrophic lateral sclerosis.^33^ Similarly, modulatory effect of urate on the risk and disease progression in MSA has been observed in male patients.^9^, ^10^ However, in this study, the association between serum urate levels and white matter disintegrity was observed in both male and female patients without significant urate by sex interaction, in spite of difference in the mean serum urate levels between sexes. These results imply that urate may be protective against white matter degeneration in MSA patients irrespective of sex. Because serum urate concentrations were typically higher in men than in women, the antioxidative effects of urate seems more likely to be seen in men.. However, some studies have reported an inverse association between urate and Parkinson’s disease risk even in female patients,^31^, ^34^ which is consistent with our results. Since estrogen has an inverse correlation with serum urate levels^35^ and a protective effect on neurodegeneration and neuroinflammation,^36^, ^37^ it is inappropriate to apply the antioxidant effect of absolute serum urate levels equally to both sexes. Future studies focusing on sex‐specific antioxidant effect of urate in MSA are warranted.

Subgroup analysis according to the clinical subtypes of MSA revealed that the correlation between urate and white matter degeneration was primarily observed in MSA patients with predominant cerebellar type. Despite the common pathological findings of oligodendroglial cytoplasmic inclusions, MSA‐P and MSA‐C are different in terms of clinical, neuropathological, and imaging characteristics.^1^ Although the mechanism that determines the clinically different subtypes in MSA remains largely unknown, we can infer from this study that oxidative stress may play a pivotal role in the pathogenesis of phenotypic heterogeneity in MSA. This result also has an implication for trial design that targets antioxidation as a disease‐modifying therapy in MSA.

Path analysis revealed that white matter disintegrity in cerebellar peduncle mediated the association of serum urate level with disease severity in MSA. Consistent with our results, a previous study has reported that patients with MSA showed a negative correlation between serum urate level and disease severity.^38^ Imaging studies using DTI have demonstrated that ataxia scale was associated with FA values in cerebellar white matter, middle cerebellar peduncle, and posterior limb of internal capsule and with MD values in cerebellar white matter in MSA‐C.^6^ These studies suggest that serum urate and white matter disintegrity are closely interacted with disease severity in MSA. Pathological studies have reported that in MSA, there are neuronal loss, deposition of glial cytoplasmic inclusion, and extensive myelin loss in medullo‐ponto‐cerebellar regions, which are interconnected by middle and inferior cerebellar peduncles.^39^ Considering the important role of oxidative stress in degeneration of oligodendrocyte,^40^ urate may engage in modulation of MSA‐related white matter degeneration, which in turn, could affect clinical manifestation of MSA. Thus, it is plausible that cerebellar peduncle may be an important anatomical substrate linking oxidative stress and disease severity in MSA.

The present study has several limitations. First, the cross‐sectional design limited to identify a causal relationship between serum urate levels and white matter disintegrity. Longitudinal study studies are required to elucidate the protective role of urate in white matter degeneration. Second, there are many risk factors associated with hyperuricemia or white matter integrity that may interfere with the assessment of independent relationship between urate and white matter integrity, such as cognitive function, smoking, diet, body mass index, and renal function. To generalize the results, a larger sample size is required, adjusting a sufficient number of important variables and strictly matching the controls. Third, we recruited MSA patients with varying disease duration and severity. Association of urate and white matter integrity according to disease duration or severity might present a different pattern of the role of urate as an antioxidant as the neurodegeneration progresses. Fourth, pathologic evaluation was not performed in all patients. Such an evaluation may help in studying the definite relationship between serum urate levels and the degree of demyelination or axonal injury.

The present study demonstrated that serum urate levels played a pivotal role in microstructural white matter disintegrity and clinical disability in patients with MSA. The results of the present study provide potential evidence that therapy targeting serum urate levels may be a possible candidate for disease‐modifying therapy in MSA.

## Conflict of Interest

The authors declare that they have no competing interests.

## Author Contributions

H.S.Y., H.K., and P.H.L. contributed to the concept and design of the study. H.S.Y., S.J.C., Y.H.L., H.K., and P.H.L. contributed to the acquisition and analysis of the data. All authors contributed to drafting the text and preparing the figures.

## Supporting information


**Method S1.** Neuroimaging acquisition.
**Method S2.** Processing of DTI data.
**Method S3.** Processing of high‐resolution T1‐weighted MR data.
**Figure S1.** Group comparison of white matter integrity (A) between MSA patients and normal controls and (B) between male and female MSA patients.
**Table S1.** Path analyses of serum urate level or DTI measures for total UMSARS score
