# Supplementary material for: Urate is closely linked to white matter integrity in multiple system atrophy
Source: Ann Clin Transl Neurol. 2020 Jun 26;7(6):1029–39. doi: 10.1002/acn3.51073 (PMC7318089; doi:10.1002/acn3.51073)
Supplement: Supplementary file 1 — Method S1. Neuroimaging acquisition. Method S2. Processing of DTI data. Method S3. Processing of high‐resolution T1‐weighted MR data. Figure S1. Group comparison of white matter integrity (A) between MSA patients and normal controls and (B) between male and female MSA patients. Table S1. Path analyses of serum urate level or DTI measures for total UMSARS score [file ACN3-7-1029-s001.docx]

**Supplementary Materials**

**Supplementary Method S1.** Neuroimaging acquisition

The magnetic resonance imaging (MRI) scans were acquired using a Philips 3T scanner (Philips Intera, Philips Medical System, Best, The Netherlands) with a SENSE head coil (SENSE factor = 2). High-resolution T1-weighted MRI volumes were acquired axially using three-dimensional gradient echo sequence with the following parameters: axial acquisition with a 224 × 246 matrix, 256 × 256 reconstructed matrix with 170 slices, 220 mm field of view; 0.859 × 0.859 × 1 mm3 voxels, TE (echo time) of 4.6 ms, TR (repetition time) of 9.8 ms, flip angle of 8°, and no slice gap. Diffusion tensor imaging (DTI) was acquired with the following parameters: axial acquisition with a 112 × 112 matrix, 2 × 2 × 2 mm3 voxels, 70 axial slices, 224 mm field of view, TE of 71 ms, TR of 7218 ms, flip angle of 90°, slice gap of 0 mm, and b-factor of 600 mm2. Diffusion-weighted images were acquired from 32 different directions with one T2-weighted b0 image**.**

**Supplementary Methods S2.** Processing of DTI data

The Functional MRI of Brain (FMRIB) Software Library (FSL) (http://www.fmrib.ox.ac.uk/fsl) was used to preprocess the DTI data. Motion artifacts and eddy current distortion were corrected by normalizing each diffusion-weighted volume to the non-diffusion weighted volume (b0) using affine registration method in the FMRIB’s Linear Image Registration Tool (FLIRT). Using a general linear-fitting algorithm, diffusion tensor matrices were generated from the sets of diffusion-weighted images. Subsequently, fractional anisotropy (FA) and mean diffusivity (MD) were calculated for each voxel according to simple linear fitting algorithm using the DTIFIT Tool (part of FSL).

The FA and MD maps of DTI pre-processing results were used for the TBSS analysis.^1^ We aligned all FA images onto a standard FMRIB58 FA template provided by the FSL software. For this process, we used a nonlinear registration algorithm implemented in the TBSS package. The FA images aligned on the FMRIB58 FA template were averaged to create a mean FA image. It was then skeletonized to represent the center of the white matter (WM) tract of the group. We chose an FA threshold value of 0.2 to exclude the voxels of gray matter (GM) or the cerebrospinal fluid (CSF). Aligned FA images from each patient were projected onto the skeleton by filling the skeleton with the highest FA values at the nearest relevant center of the fiber tracts. MD images were processed by implementing the FA nonlinear registration and projecting them onto the skeleton using methods identical to those derived from the original FA data.

**Supplementary Method S3.** Processing of high-resolution T1-weighted MR data

We visually validated the quality of T1 weighted MRI before extracting cortical surface model and cortical thickness. Structural MR images were registered into a standardized stereotaxic space using linear transformation.^2^ The N3 algorithm was used to correct images for intensity non-uniformity resulting from inhomogeneity in the magnetic field.^3^ The non-brain tissues of registered and corrected images were removed using Brain Extraction Tool (BET)^4^ and then classified into GM, WM, CSF, and background using the Intensity-Normalized Stereotaxic Environment for Classification of Tissues (INSECT) algorithm.^5^ The surfaces of the inner and outer cortices which consisted of 40,962 vertices were automatically extracted using the Constrained Laplacian-based Automated Segmentation with Proximities (CLASP) algorithm, which reconstructs the inner cortical surface by deforming a spherical mesh onto the WM/GM boundary and then expanding the deformable model to the GM/CSF boundary.^6,7^ Cortical thickness was defined using the t-link method, which captures the Euclidean distance between the linked vertices of the inner and outer cortical surfaces.^6,8^ Each individual cortical thickness map was smoothed with 20mm full-width half-maximum Gaussian smoothing kernel to increase the signal to noise ratio,^8,9^ and aligned to unbiased interative surface template using vertex-wise sphere-to-sphere nonlinear surface registration.^10,11^

**
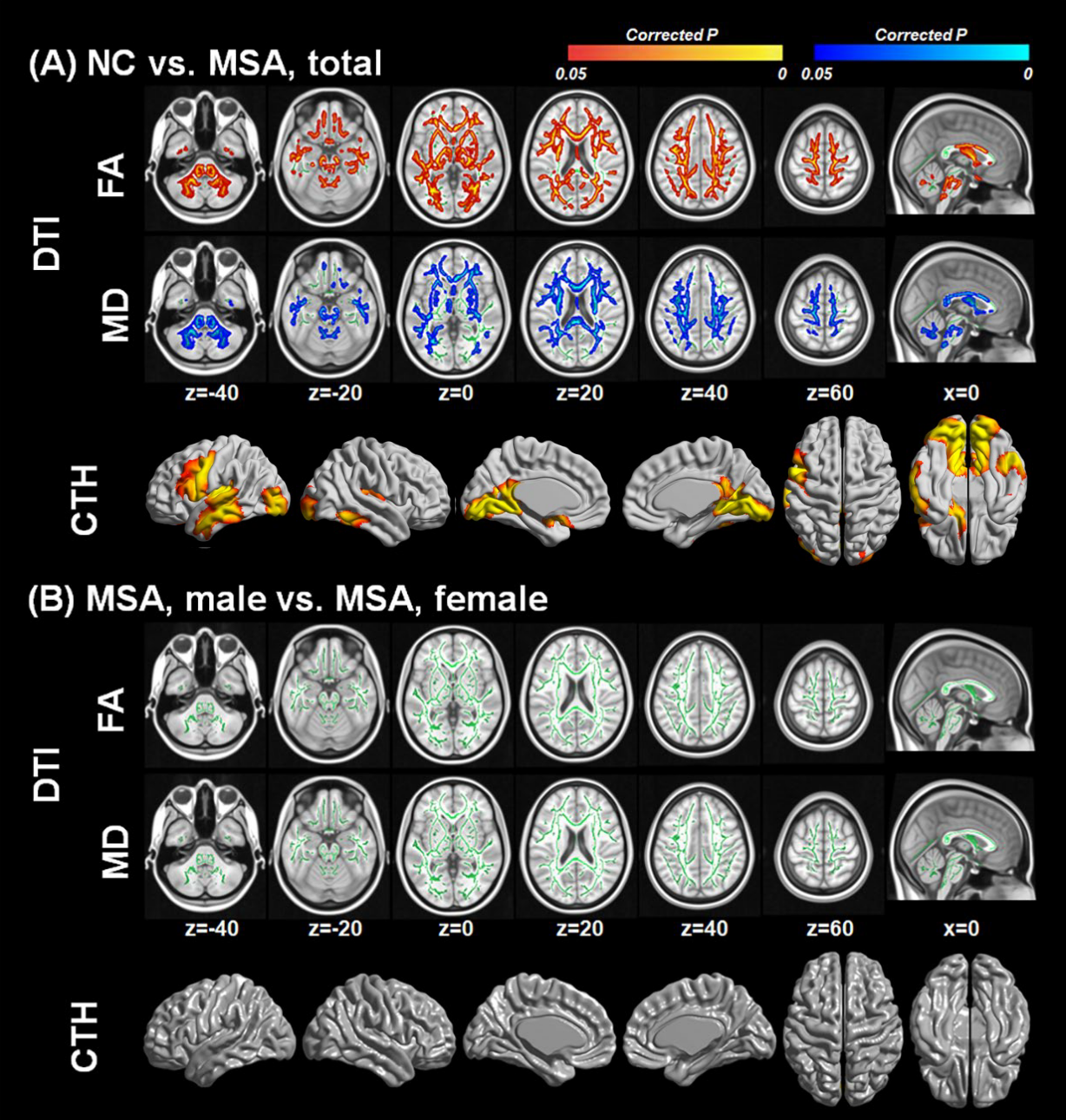
**

**Supplementary Figure S1.** Group comparison of white matter integrity (A) between MSA patients and normal controls and (B) between male and female MSA patients. Age and sex were adjusted in the comparison between MSA patients and normal controls, while age and disease duration was adjusted in the comparison between male and female MSA patients. Corrections for multiple comparisons were performed using family-wise error rate in diffusion tensor imaging analysis and random field theory in cortical thickness analysis respectively. Red to yellow color in corrected *P*-map indicates white matter regions where the former group has higher value than the latter group. Blue color in corrected *P*-map indicates white matter regions where the former group has a lower value than the latter group. The threshold was set at corrected *P* < 0.05. The brain images were displayed in neurological convention.

**Supplementary Table S1.** Path analyses of serum urate level or DTI measures for total UMSARS score

|  | DTI measures | | | Total UMSARS score | | |
| --- | --- | --- | --- | --- | --- | --- |
| Predictors | Beta | SE | *P* value | Beta | SE | *P* value |
| Serum urate level | −0.25 | 0.10 | 0.027 | -0.15 | 0.11 | 0.399 |
| MD in middle cerebellar peduncle |  |  |  | 0.33 | 0.13 | 0.040 |
| Serum urate level | −0.30 | 0.09 | 0.010 | -0.06 | 0.12 | 0.780 |
| MD in inferior cerebellar peduncle |  |  |  | 0.37 | 0.14 | 0.035 |

Data are results of path analyses of total UMSARS score using serum urate level as a predictor and DTI measures as a mediator after controlling for age and sex. Among white matter regions, those showing significant association with total UMSARS score (middle cerebellar peduncle and inferior cerebellar peduncle) were selected. These models showed good fit to total UMSARS score [for middle cerebellar peduncle, χ^2^ = 8.087, degree of freedom (dF) = 10, *P* = 0.620, Confirmatory Fit Index (CFI) = > 0.999, Root Mean Square Error of Approximation (RMSEA) = < 0.001; for inferior cerebellar peduncle, χ^2^ = 10.381, dF = 11, *P* = 0.496, CFI = > 0.999, RMSEA = < 0.001].

**References**

1. Smith SM, Jenkinson M, Johansen-Berg H, et al. Tract-based spatial statistics: voxelwise analysis of multi-subject diffusion data. Neuroimage 2006;31:1487-1505.

2. Collins DL, Neelin P, Peters TM, Evans AC. Automatic 3D intersubject registration of MR volumetric data in standardized Talairach space. J Comput Assist Tomogr 1994;18:192-205.

3. Sled JG, Zijdenbos AP, Evans AC. A nonparametric method for automatic correction of intensity nonuniformity in MRI data. IEEE Trans Med Imaging 1998;17:87-97.

4. Smith SM. Fast robust automated brain extraction. Hum Brain Mapp 2002;17:143-155.

5. Zijdenbos A, Evans A, Riahi F, Sled J, Chui J, Kollokian V. Automatic quantification of multiple sclerosis lesion volume using stereotaxic space. Visualization Biomed Comput 1996;1131:439-448.

6. MacDonald D, Kabani N, Avis D, Evans AC. Automated 3-D extraction of inner and outer surfaces of cerebral cortex from MRI. Neuroimage 2000;12:340-356.

7. Kim JS, Singh V, Lee JK, et al. Automated 3-D extraction and evaluation of the inner and outer cortical surfaces using a Laplacian map and partial volume effect classification. Neuroimage 2005;27:210-221.

8. Im K, Lee JM, Lee J, et al. Gender difference analysis of cortical thickness in healthy young adults with surface-based methods. Neuroimage 2006;31:31-38.

9. Lerch JP, Evans AC. Cortical thickness analysis examined through power analysis and a population simulation. Neuroimage 2005;24:163-173.

10. Robbins S, Evans AC, Collins DL, Whitesides S. Tuning and comparing spatial normalization methods. Med Image Anal 2004;8:311-323.

11. Lyttelton O, Boucher M, Robbins S, Evans A. An unbiased iterative group registration template for cortical surface analysis. Neuroimage 2007;34:1535-1544.
